# Latent autoimmune diabetes in adults: a focus on β-cell protection and therapy

**DOI:** 10.3389/fendo.2022.959011

**Published:** 2022-08-05

**Authors:** Wenfeng Yin, Shuoming Luo, Zilin Xiao, Ziwei Zhang, Bingwen Liu, Zhiguang Zhou

**Affiliations:** National Clinical Research Center for Metabolic Diseases, Key Laboratory of Diabetes Immunology (Central South University), Ministry of Education, and Department of Metabolism and Endocrinology, The Second Xiangya Hospital of Central South University, Changsha, China

**Keywords:** LADA (Latent Autoimmune Diabetes in Adults), autoimmune, β-cell function, type 1 diabetes, heterogeneous disease

## Abstract

Latent autoimmune diabetes in adults (LADA) is a heterogeneous disease sharing some phenotypic, genetic, and immunological features with both type 1 and 2 diabetes. Patients with LADA have a relatively slow autoimmune process and more residual islet β-cell function at onset, allowing a time window to protect residual islet β cells and delay or inhibit disease progression. It is crucial to discover various heterogeneous factors affecting islet β-cell function for precise LADA therapy. In this review, we first describe the natural history of LADA. Thereafter, we summarize β-cell function-related heterogeneous factors in LADA, including the age of onset, body mass index, genetic background, and immune, lifestyle, and environmental factors. In parallel, we evaluate the impact of current hypoglycemic agents and immune intervention therapies for islet β-cell protection. Finally, we discuss the opportunities and challenges of LADA treatment from the perspective of islet β-cell function protection.

## 1 Introduction

Diabetes is generally regarded as a disease spectrum ranging from classic insulin-resistant type 2 diabetes to classic insulin-dependent type 1 diabetes (1). Latent autoimmune diabetes in adults (LADA) is a heterogeneous form of diabetes with common clinical features of both type 1 and 2 diabetes (2, 3). It is also known as type 1.5 diabetes or slowly progressive insulin-dependent type 1 diabetes. LADA accounts for approximately 2%–14% of all diabetes patients (4). Latest data have shown that the number of LADA among the Chinese population has exceeded 10 million (5, 6), being the largest number of LADA patients in the world.

There is no universal agreement on LADA classification, diagnosis, and management. It is called slowly evolving immune-mediated diabetes and is classified as a type of hybrid forms of diabetes in the Classification of Diabetes Mellitus 2019 of the World Health Organization (7). However, it is also classified as a subtype of type 1 diabetes because of its autoimmune destruction of islet β cells according to the Diagnosis and Classification of Diabetes Mellitus of the American Diabetes Association in 2022 (8). Currently, the exact underlying mechanisms of LADA are poorly understood, and specific etiological treatment of LADA remains an issue.

Loss of islet β-cell function due to autoimmunity is the key factor in LADA occurrence and progression, and individuals with LADA have more residual islet β cells at onset compared with classic type 1 diabetes (9, 10). After diagnosis, the ability to retain residual β-cell function is heterogeneous, being influenced by the age of onset, genetics, and immune mechanisms. A comprehensive understanding of the influencing factors and different protection methods of islet β-cell function is clinically significant to further explore how to effectively protect islet β cells and delay disease progression. Herein, we summarize related heterogeneous factors for islet β-cell protection. Then, we evaluate the impact of current hypoglycemic agents and immune intervention therapies for islet β cells. Finally, we discuss the opportunities and challenges of LADA treatment from the perspective of islet β-cell function protection.

## 2 Natural history of LADA

**Figure 1:**
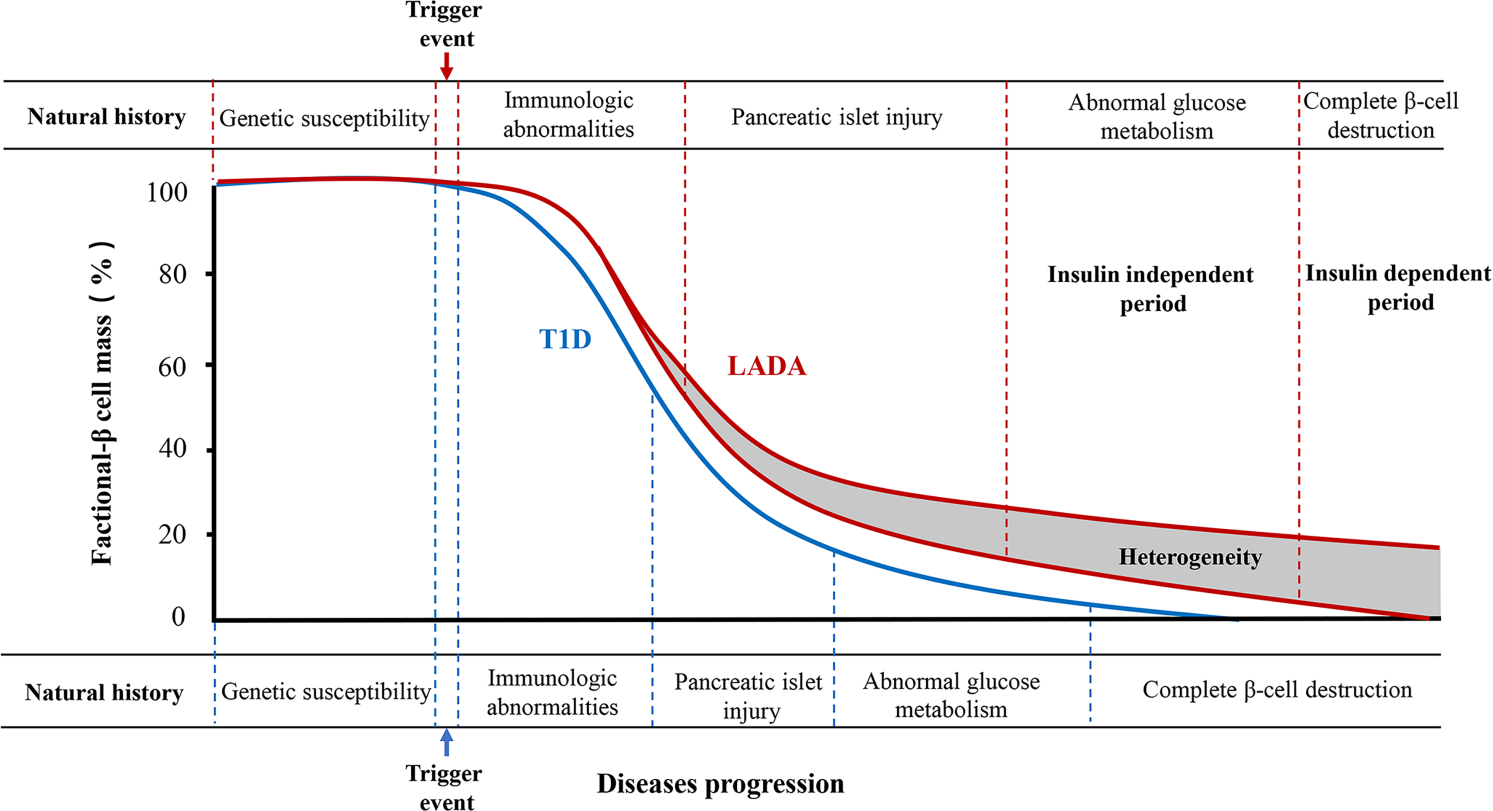
The natural history of type 1 diabetes and LADA. Type 1 diabetes can be divided into six stages. LADA has a similar natural course to type 1 diabetes as its autoimmune subtype. However, the rate of decline of pancreatic β-cell function is lower than that of type 1 diabetes. Moreover, the natural history of most LADA patients can be divided into insulin-independent and insulin-dependent periods according to the degree of pancreatic β-cell destruction. There is great heterogeneity in the progression to insulin dependence in patients with LADA, which is associated with the age of onset, body mass index, genetic background, and immune, lifestyle, and environmental factors.

The traditional natural history of type 1 diabetes can conceptually be divided into six stages. The stages are genetic predisposition; an action of the precipitating event; overt immunologic abnormalities with normal insulin release; progressive loss of insulin release with normal glucose level; overt diabetes with present C-peptide; and complete β-cell destruction (no C-peptide) (11). LADA, as a subtype of type 1 diabetes, has a similar natural course (**Figure 1**). However, because of the heterogeneity of β-cell function in LADA patients, the course has two special features. One, pancreatic β-cell function declines faster in LADA patients than in those with type 2 diabetes but slower than that of classic type 1 diabetes (12). Another, several previous studies have shown that levels of C-peptide in LADA patients continued to decline rapidly when followed up for 3 or 6 years (13, 14). The decline pattern of β-cell function in LADA was biphasic, showing an initial rapid progression followed by a stable mode (15).

The clinical course of most LADA patients can be divided into insulin-independent and insulin-dependent stages (16). The insulin-independent stage is the early clinical stage of LADA, with similar clinical features to type 2 diabetes. No typical clinical symptoms are present in this stage. Oral hypoglycemic agents are capable of controlling blood glucose at this stage. Due to presence of heterogeneity, there are LADA patients whose diabetes can also be controlled with diet alone, weight loss, and an exercise program, without any oral medications. These are stable forms that can last for decades and require insulin therapy only very late, when LADA patients’ islet β-cell function becomes significantly impaired and leads to diabetic ketosis or acidosis. Additionally, insulin therapy becomes necessary, and patients thereafter enter the insulin-dependent stage.

## 3 Heterogeneity of LADA and islet β-cell function

LADA exhibits significant clinical heterogeneity. Patients with LADA have different clinical, immunological, and genetic characteristics when compared with type 1 and 2 diabetes. The main parameter differences among them are summarized in **Table 1** (2, 14). These factors of clinical heterogeneity are also related to islet β-cell function in patients with LADA.

**Table 1:** Differences in key parameters between type 1 diabetes, LADA, and type 2 diabetes.

Disease feature	Type 2 diabetes	LADA	Type 1 diabetes
Age of onset	Mostly at adulthood	Older than 30 years	Most commonly occurs in children, but it may occur at any age
Body mass index	Overweight/obesity	Normal/overweight	Underweight/normal
HLA susceptibility	No change	Increased	Considerably increased
Number of islet autoantibodies	None	Increased	Considerably increased
C-peptide levels	High	Low	Considerably low
Insulin dependence	Mostly late (8-10 years)	Early (after 6 months)	Always

### 3.1 Age of onset

LADA patients with different ages of onset show different clinical features and islet β-cell function. Accumulating evidence shows that individuals with LADA that are younger at disease onset have a lower level of insulin resistance and worse residual β-cell function than those with an older age of onset (16, 17). A recent study has indicated that LADA patients with the old age of onset (≥60 years) tend to have higher fasting C-peptide levels and 2-h postprandial C-peptide levels compared with those with a young age of onset (<60 years) (17). Therefore, the age of LADA onset may be associated with islet β-cell destruction, with patients with the old age of onset having more residual β-cell mass and better islet β-cell function.

### 3.2 Body mass index

Overweight/obesity is a risk indicator for patients with LADA (18, 19). Data indicate that 66% of LADA falls into the overweight/obesity category (20). Although patients with LADA are leaner compared with type 2 diabetes patients, LADA-China study has shown that the median body mass index (BMI) was 24.5 kg/m^2^ in LADA patients, and the proportion of overweight and obese patients was up to 41.7% (9). Emerging evidence has demonstrated a significant correlation between BMI and islet β-cell demise and dysfunction. Compared with classic type 1 diabetes, LADA patients are more obese and have a larger waist circumference, lower low-density lipoprotein cholesterol levels, and more residual islet β cells (21). Moreover, obese LADA patients show a lower frequency of insulin dependence and have better β-cell function (22, 23). The reason why β-cell depletion is slower in LADA patients with higher waist circumference is unclear. Perhaps, adipose tissue-induced insulin resistance increases fasting glucose levels earlier compared to lean LADA patients, leading to prompt management of the disease (24). Additionally, diabetic obese mice with leptin receptor knockout causing hyperleptinemia show that obesity may delay the onset of insulin dependence (25). This concept diverges from the “accelerator hypothesis,” which refers to the onset of the autoimmune reaction itself. The “accelerator hypothesis” argues that intrinsic nature, insulin resistance, and autoimmunity can accelerate islet β-cell loss through apoptosis in diabetes patients (26). Obesity may enhance insulin resistance and islet autoantigen exposure, contributing to the initiation of autoimmune destructive processes (27).

### 3.3 Genetic background

Susceptibility genes, as a predictor and player of the disease, play a vital role in the islet β-cell failure in diabetes. Recent studies have verified the genetic overlap between LADA and both type 1 and 2 diabetes (28). Human leukocyte antigen (HLA) class II genes are the most susceptible genes in LADA patients and can mediate the autoimmune response (29, 30). Most type 1 diabetes-associated HLA haplotypes also confer LADA susceptibility (31). A study has revealed that *DR9/DR9* is the Chinese-specific LADA risk genotype, while *DR3/DR4* is the major risk factor in Caucasians (32). A study of Caucasians has indicated that the *DRB1/DQB1* genotype in LADA was associated with the age of onset, and LADA patients with HLA *DRB1* and *DQB1* developed the disease at a younger age and had severe islet β-cell function (33). Additionally, previous studies have shown that *HLA-DQB1* genotypes are most frequent in early-onset classic type 1 diabetes patients who were diagnosed at <20 years, followed by late-onset classic type 1 diabetes patients who were diagnosed at >35 years and LADA patients (34). This further suggests that HLA genes are related to islet β-cell function. Japanese LADA subjects with insulin dependence more often had DRB1*0405-DQB1*0401, DRB1*0802-DQB1*0302, and DRB1*0901-DQB1*0303 haplotypes, whereas only the DRB1*0405-DQB1*0401 haplotype occurred more often in non-insulin-dependence LADA patients, additionally reinforcing the stated point (35).

Some non-HLA genes, including type 1 diabetes-associated variants and type 2 diabetes-associated variants, also have a role in LADA. Insulin (INS) (36), cytotoxic T lymphocyte-associated protein 4 (CTLA4), and SH2B adapter protein 3 (SH2B3) are implicated in LADA pathogenesis (37). *PFKFB3* has been linked to immune and metabolic diabetes (30). A previous study has indicated that the *PFKFB3* product can regulate glycolysis and insulin pathway (38). A further experiment in mice has revealed that insulin resistance exacerbation and increased adipose tissue inflammation occurred if *PFKFB3* expression in adipose tissue was disturbed (39). Additionally, targeted overexpression of the gene can protect against diet-induced insulin resistance and inflammatory responses (40). Moreover, genome-scale *in vivo* CRISPR screening has identified *RNLS* as a target for β-cell protection in type 1 diabetes. By deleting *RNLS*, β cells can become resistant to autoimmune killing in type 1 diabetes mice (41). However, its specific function in LADA remains unclear. Briefly, it can be concluded that LADA patients share a genetic predisposition with type 1 and 2 diabetes.

### 3.4 Immune mechanisms

LADA is a heterogeneous disease characterized by immune-mediated β-cell destruction. In recent years, multiple studies have reported that innate, cellular, and humoral immunity plays an important role in LADA pathogenesis (42–44).

#### 3.4.1 Innate immunity

A cross talk is believed to exist between β cells and various immune cells from adaptive and innate immunity during the initiation and progression of autoimmune diabetes (45). In LADA occurrence and progression, macrophages, natural killer (NK) cells, dendritic cells (DCs), and neutrophils (NEs) play a crucial role. First, macrophages are present in the pancreas of LADA patients and a rat model, and an increase in interleukin-1β (IL-1β) has been able to recruit more macrophages to infiltrate the pancreas, leading to apoptosis of β cells (46). Other studies have also shown that macrophages infiltrate pancreatic islets and participate in the destruction of β cells (47, 48). A further study has shown that macrophages can produce more IL-1β, and IL-1β may contribute to β-cell destruction (49).

Second, NK cells may also be involved in LADA development. Wang et al. (50) have found a higher number of inducible interferon (IFN)-γ ^(+)^ NK cells in newly diagnosed Chinese LADA patients compared to controls. IFN-γ released by NK cells may promote LADA development by affecting islet β cells. The role of NK cells in LADA is not well understood, with some studies reporting a decrease in the number of NK cells, while other studies have indicated an increase in the number of NK cells (51, 52). In a Caucasian population with LADA, the number of CD3^-^CD56^+^ NK cells has decreased, unlike that in healthy individuals (52), whereas in Chinese LADA patients, the number of activated NKp46^(+)^ NK cells has significantly increased (50). Interestingly, the expression of NKG2D, a surface-activated receptor for NK cells, has been reduced in type 1 diabetes patients and an animal model (53, 54). In contrast, in patients with LADA, NKG2D expression has been increased, while the expression of killer cell immunoglobulin-like receptor 3DL1 has been decreased (52). This might imply a distinction between LADA patients with slower progression of β-cell injury (52).

DCs are involved in pancreatitis along with macrophages and activate CD4^+^ T cells *via* IL-12 secretion (55). Evidence has shown that the proportion of CD123^-^CD11c^+^ DCs is lower in patients with LADA than that in patients with type 1 diabetes, implying a slower progression of LADA, unlike in acute type 1 diabetes patients (56). However, to date, few studies have been performed on the importance of DCs in LADA.

Finally, NE counts have shown a gradual increase from type 1 diabetes and LADA to type 2 diabetes and correlated with the number and level of islet β-cell autoantibodies (57). Data have shown that patients positive for glutamic acid decarboxylase antibodies (GADA), protein tyrosine phosphatase 2 antibodies (IA2A), and selective zin transporter 8 (ZnT8A) all had minimal NE counts, suggesting an association between NE and autoimmune dysfunction and β-cell failure in diabetic patients (57). In conclusion, although the exact mechanism of β-cell failure is still unknown, the cross talk between immune cells is slowly becoming elucidated.

#### 3.4.2 Cellular immunity

Insulitis is a hallmark of β-cell immune-mediated dysfunction and is characterized by various immune cell infiltrates, consisting of CD8+ cytotoxic T cells, CD4^+^ T cells, and B cells (58, 59). Insulitis exists in the pancreas of LADA patients, as indicated by pancreatic scintigraphy (60), and it has been verified in isolated pancreatic tissue and LADA animal model (46, 61). Rolandsson et al. (62) noted that type 2 diabetes patients with antigen-specific reactive T cells and no autoantibodies were termed as T-LADA (Ab-T+). T-LADA (Ab-T+) patients have worse β-cell function distinct from classic type 2 diabetes (Ab-T-) (63). The latest study also indicated that T cell-mediated autoimmunity plays an important role in β-cell destruction and worse glycemic control (64), implying that altered proportions and functional defects of T-cell subsets are an important cause of autoimmunity in LADA. Studies found that decreased regulatory T cells in LADA mediate β-cell damage (65). Further experiments have revealed that the expression level of forkhead box protein 3 (FOXP3) is downregulated in peripheral CD4^+^ T cells, and this FOXP3 expression is regulated by STAT3-mediated epigenetic silencing through HDAC5 and DNA methyltransferase 3b (66, 67). In addition to T-cell subsets, B-cell subsets also have an alteration, in which the size of the marginal zone B is increased while the number of follicular B and IL-10-producing regulatory B (B10) cells is decreased. This change has been associated with the loss of self-tolerance and the destruction of islet β cells (68). A previous study has suggested that B10 cells can exert anti-inflammatory effects and maintain peripheral tolerance by secreting IL-10 in type 1 diabetes patients (69).

#### 3.4.3 Humoral immunity

Humoral immunity is primarily reflected in the presence of islet autoantibodies in the serum of patients with LADA. In fact, autoimmunity to islet β cells precedes the onset of LADA by several years. A prospective study has verified this phenomenon in nearly 60% of LADA patients (70). GADA, as one of the most potent autoantibodies and sensitive diagnostic markers, is involved in β cell-specific autoimmunity (71). The discussion on whether GADA levels can predict the functional exhaustion of β cell has lasted for years without reaching definitive conclusions. Some believe that GADA antibodies have high predictive power (72), while others do not (73). Furthermore, a transient increase in circulating GADA levels may result from body weight gain or β-cell stress, not necessarily indicating an underlying ongoing autoimmune process. Quantitative data on antibody levels should make it easier to decide whether patients have true LADA or a mere surge in antibody levels due to metabolic decompensation. Additionally, the affinity and identifying specific epitopes of islet autoantibodies help improve the prediction of insulin deficiency (74). The electrochemiluminescence (ECL) assay is a promising islet autoantibody detection method that can precisely discriminate between high-affinity and high-risk specific autoantibodies, leading to an earlier identification of islet autoimmunity initiation before clinically evident diabetes (75). According to ECL analysis, ECL-GAD65 antibody-positive participants resemble type 1 diabetes patients with slender body size and poor β-cell function, whereas ECL-GAD65 antibody-negative patients have a similar phenotype to type 2 diabetes individuals with good β-cell mass and a slow rate of insulin insufficiency (74). The presence of IA-2A is also effective in predicting the future need for insulin therapy (76). A report indicated that among seven IA-2 constructs, the IA-2 (256–760) fragment was analyzed as the most sensitive marker for detecting humoral IA-2 immunoreactivity in patients with LADA (77). Moreover, as verified in an animal model, the reduced activity of pancreatic β cells ZnT8 can contribute to impaired β-cell function and accelerate diabetes related to islet amyloidosis (78). Recently, a new autoantibody of LADA, tetraspanin 7 autoantibodies (TSPAN7A), has been investigated. TSPAN7A is a valid islet autoantibody for use in East Asian populations suffering from LADA and can discriminate against individuals with LADA who have a lower β-cell function after disease progression (79).

### 3.5 Environmental factors

Environmental factors may also contribute to LADA occurrence and progression. Numerous studies have indicated that physical activity, smoking, drinking, sweetened beverages, coffee intake, viruses, and gut microbiota are related to disorders in glucose metabolism, insulin sensitivity, and autoimmune destruction of LADA (80–83). High physical activity can regulate metabolism and increase immunity (80). Individuals who exercise daily have a three-fold lower risk of developing LADA compared to those who exercise less than once a week (81). Notably, the association between smoking and LADA incidence has been contradictory. A 22-year follow-up study found that heavy smoking can suppress autoimmunity in a Norwegian population with LADA (84), while some data from a Swedish case–control study indicated that heavy smoking increases the risk of LADA compared with never-smokers who have high levels of homeostatic model assessment of insulin resistance and β-cell function and lower levels of GADA (85). Additionally, certain evidence has suggested that smoking or nicotine has a biphasic action, which depends on patients’ different smoking statuses (86, 87). Further studies have indicated that former and current smoking is associated with high or low islet β-cell function, respectively (88).

Recently, an emerging study has demonstrated a significant link between gut microbiota and autoimmune targeting of islet β cells (83). LADA patients exhibit various gut microbiota and metabolic profiles compared with healthy subjects and classic type 1 and 2 diabetes patients. Furthermore, data have suggested that patients with LADA have a decrease in short-chain fatty acid (SCFA)-producing bacteria. SCFA-producing bacteria can regulate intestinal hormones, increase insulin sensitivity, and attenuate islet β-cell destruction (89, 90). Additionally, acetate and butyrate derived from non-obese diabetic mice gut microbes have been demonstrated to serve as protective factors to inhibit insulitis development. In contrast, acetate and butyrate in the diet decrease the proportion of autoreactive T cells, increase regulatory T-cell counts, enhance the function of regulatory T cells, and boost gut integrity (91). A further study has shown that the transfer of microbiota to pancreatic lymph nodes triggered the intracellular protein receptor nucleotide-binding oligomerization domain-containing 2 (NOD2) activation and contributed to the onset of type 1 diabetes (92). Remarkably, this suggests that gut dysbiosis in patients with autoimmune diabetes may contribute to the onset and progression of the disease, but this requires further high-quality evidence to confirm it.

## 4 Hypoglycemic agents and islet β-cell protection

Currently, for LADA drug treatment, some hypoglycemic agents can prolong the duration of the insulin-independent stage progression to the insulin-dependent stage. Achieving strict glycemic management is the basis of preventing or postponing preserved β-cell destruction and inhibiting the development of diabetic complications in LADA patients.

### 4.1 Insulin

Certain studies have reported that insulin therapy is rational and essential for intervention in progressive β-cell deterioration in LADA patients, especially in patients with the early onset and more preserved β-cell function (93–95). The possible reason for protecting β-cell function using insulin in LADA patients may be due to a decrease in antigen expression and autoimmune attack on β cells as a result of exogenous insulin inhibition of β-cell activity (94). Furthermore, exogenous insulin may lead to immune tolerance and inhibit subsequent immune pathways, thereby protecting residual β cells (94). Additionally, exogenous insulin prevents the accumulation of amylin-positive amyloid in β cells, which is considered one of the most important pathogenic mechanisms of β-cell failure (94). One study reported that therapy with intensive insulin in newly diagnosed type 2 diabetic patients has a great significance for the recovery and maintenance of β-cell function (96). We speculate that intensive insulin therapy can protect islet β-cell function in LADA patients. However, to date, studies on insulin intensification in patients with LADA have not been reported.

### 4.2 Insulin sensitizer

Metformin, an insulin sensitizer, is the most commonly used hypoglycemic drug. Patients with LADA who have not yet progressed to the insulin-dependent stage often utilize metformin to control their blood glucose levels. However, it has been shown that metformin does not delay or halt the progressive deterioration of β-cell function in youth with impaired glucose tolerance or newly diagnosed type 2 diabetes (97). In patients with LADA, this is unclear, and further studies are needed.

Thiazolidinediones (TZDs), as a stronger insulin sensitizer, have anti-inflammatory activity on β cells and can increase their survival during the non-insulin-dependent stage of LADA. For example, Brooks-Worrell et al. (98) treated T-LADA patients with rosiglitazone for 3 years and found that the levels of IFN-γ and IL-12 secreted by autoreactive T cells in the intervention group were lower than those in the control group, suggesting that rosiglitazone can delay islet failure by inhibiting autoreactive T cells. Moreover, clinical studies in China showed that rosiglitazone alone or in combination with insulin can maintain C-peptide levels and protect islet β-cell function in LADA patients (99). In animal models, TZDs have protected the structure and tissue integrity of pancreatic islet cells, ameliorated oxidative stress, reduced apoptosis stimulation, and promoted the proliferation of islet β cells, thereby improving insulin secretion and regulating blood glucose levels (71). Regretfully, the side effects of the cardiovascular risks of this drug have extremely limited its use in patients with LADA.

### 4.3 Dipeptidyl-peptidase-4 inhibitors

Dipeptidyl-peptidase-4 inhibitors (DPP-4is) have exhibited a promising role in β-cell function protection in LADA patients. A randomized controlled trial has observed the changes in T-cell phenotype, downregulation of messenger RNA expression, and improved glycemic control in LADA patients after 12 months of treatment with sitagliptin (100). Similarly, other studies have concluded that saxagliptin increases islet β-cell function in patients with LADA (101–103). Moreover, recent studies have shown that saxagliptin combined with vitamin D has a beneficial effect on islet β cells (104, 105). Mechanistically, DPP-4is may influence the control of glucose metabolism in patients with LADA. Further, DPP-4is increase glucagon-like peptide-1 levels and inhibit glucagon levels, thereby boosting insulin secretion after glucose loading through the activation of DPP-4 receptors. Additionally, DPP-4 receptors have also been expressed on the surface of T lymphocytes, and studies have suggested that they are associated with immune regulation, which might have a significant role in delaying and stopping β-cell immune destruction in LADA (101, 106–108).

### 4.4 Glucagon‐like peptide-1 receptor agonists

Glucagon-like peptide-1 receptor agonists (GLP-1RAs), including liraglutide and dulaglutide, have been approved to treat type 2 diabetes and obesity. They elicit robust improvements in glycemic control and weight loss, combined with cardioprotection in individuals at risk of or with preexisting cardiovascular disease (109). GLP-1RAs seem to have a potential role in the treatment of patients with overweight or obese LADA. A *post-hoc* analysis of the AWARD-2, -4, and -5 trials has found that dulaglutide can reduce glycated hemoglobin (HbA1c) levels in patients with LADA (110). Recently, liraglutide combined with an anti-IL-21 antibody has been shown to preserve islet β cells in adults with recent-onset type 1 diabetes (111). A further study has found that continuous infusion of GLP-1RA in non-obese diabetic mice can reduce β-cell apoptosis, promote β-cell regeneration, and delay disease development (112). *In vitro*, GLP-1RA can suppress cell apoptosis and enhance glucose response in freshly isolated human islets (113). However, whether GLP-1RAs can preserve islet β-cell function in LADA patients requires further confirmation by a randomized controlled clinical trial.

### 4.5 Sodium-glucose cotransporter 2 inhibitors

Sodium-glucose cotransporter 2 inhibitors (SGLT2is) are a new class of antidiabetic drugs. Research has clearly demonstrated that SGLT2is, such as empagliflozin, canagliflozin, and dapagliflozin, have pleiotropic effects in preventing cardiovascular and chronic kidney diseases beyond their favorable impact on hyperglycemia (114). Studies have reported that SGLT2is can decrease HbA1c levels in patients with type 1 diabetes, and the European Union has approved SGLT2is for the treatment of adult type 1 diabetes patients with poor blood glucose control and obesity (115, 116). However, the roles of SGLT2is in LADA have not been well studied. Recent data have suggested that the cardiorenal protective properties of these new therapies are present even in people without diabetes; thus, the extrapolation of their results on LADA individuals seems reasonable (117). SGLT2is and GLP-1RAs should be considered for the management of people with preserved insulin production and at high cardiovascular risk. The risk of diabetic ketoacidosis with SGLT2i yet requires increased vigilance by clinicians.

## 5 Immune intervention and islet β-cell protection

Several immune interventions, as an emerging and promising therapeutic approach, aimed to counteract autoimmune responses against β cells and preserve β-cell function are currently being investigated. Glutamic acid decarboxylase (GAD) is a major autoantigen in the process leading to LADA with both a clear cell-mediated immune response to GAD and autoantibodies to GAD. Administration of the GAD65 isoform can prevent autoimmune destruction of pancreatic β cells in non-obese diabetic mice and the subsequent need for exogenous insulin replacement (118–124). In phase I and II studies, an alum-formulated vaccine (Diamyd) has shown to be safe, and in a dose-finding study in LADA patients, 20 mcg of Diamyd has been given subcutaneously 1 month apart, indicating the preservation of residual insulin secretion (125, 126). Additionally, a double-blind, multicenter study has found a good safety and tolerability profile for multiple subcutaneous doses of HSP60 peptide (DiaPep277) in LADA patients (71). With this promising background, further studies are coming.

There is increasing attention to the non-mineral metabolism function of vitamin D. Evidence has shown that the vitamin D system may be involved in autoimmunity pathogenesis (127). Our previous research has shown the protective effects of 1-alpha-hydroxyvitamin D3 on residual β-cell function in patients with LADA (128). The use of immunotherapeutic agents in a combination therapy appears to be a valid approach to obtaining better results in terms of β-cell mass and function preservation. DPP-4is combined with vitamin D3 can delay the loss of β cells and improve endogenous insulin production in patients with new-onset type 1 diabetes and LADA (104, 129, 130). The main findings on the use of combination therapy with vitamin D and DPP-4is in patients with autoimmune diabetes have been summarized elsewhere (131). Vitamin D may boost anti-inflammatory response, enhance immunomodulatory effects, induce immune tolerance, and potentially stimulate insulin synthesis and secretion. Additionally, DPP-4is have been shown to develop similar actions on the innate and adaptive immune systems. The co-administration of both vitamin D and DPP-4is may substantially strengthen the efficacy of each compound as immunomodulators.

Over the past two decades, research has identified multiple immune cell types and soluble factors destroying insulin-producing β cells in type 1 diabetes. Effective immunotherapies to treat type 1 diabetes are currently under development. Bluestone et al. (132) have summarized current results for targeting complementary nodes in immunotherapies that have shown efficacy in patients with type 1 diabetes, as well as additional efforts in next-generation immune therapeutics. These immune therapeutics included cytokine antagonists, cytokine agonists, T-cell activation inhibitors, T effector cell depletion and exhaustion therapy, checkpoint agonists, regulatory cell-based therapy, B-cell antagonists, microbiome therapy, and autoantigen therapy. Whether these immunotherapies can be used for LADA and their effect of protecting β-cell function are questions expected to be answered in future research. LADA represents an ideal model for exploring immunotherapy of autoimmunity and β-cell function because it is typically associated with a lower and less severe immune-mediated β-cell destruction compared to type 1 diabetes. Therefore, LADA offers a wider window to test immune interventions that may slow down a β-cell failure.

## 6 Conclusions and future perspectives

**Figure 2:**
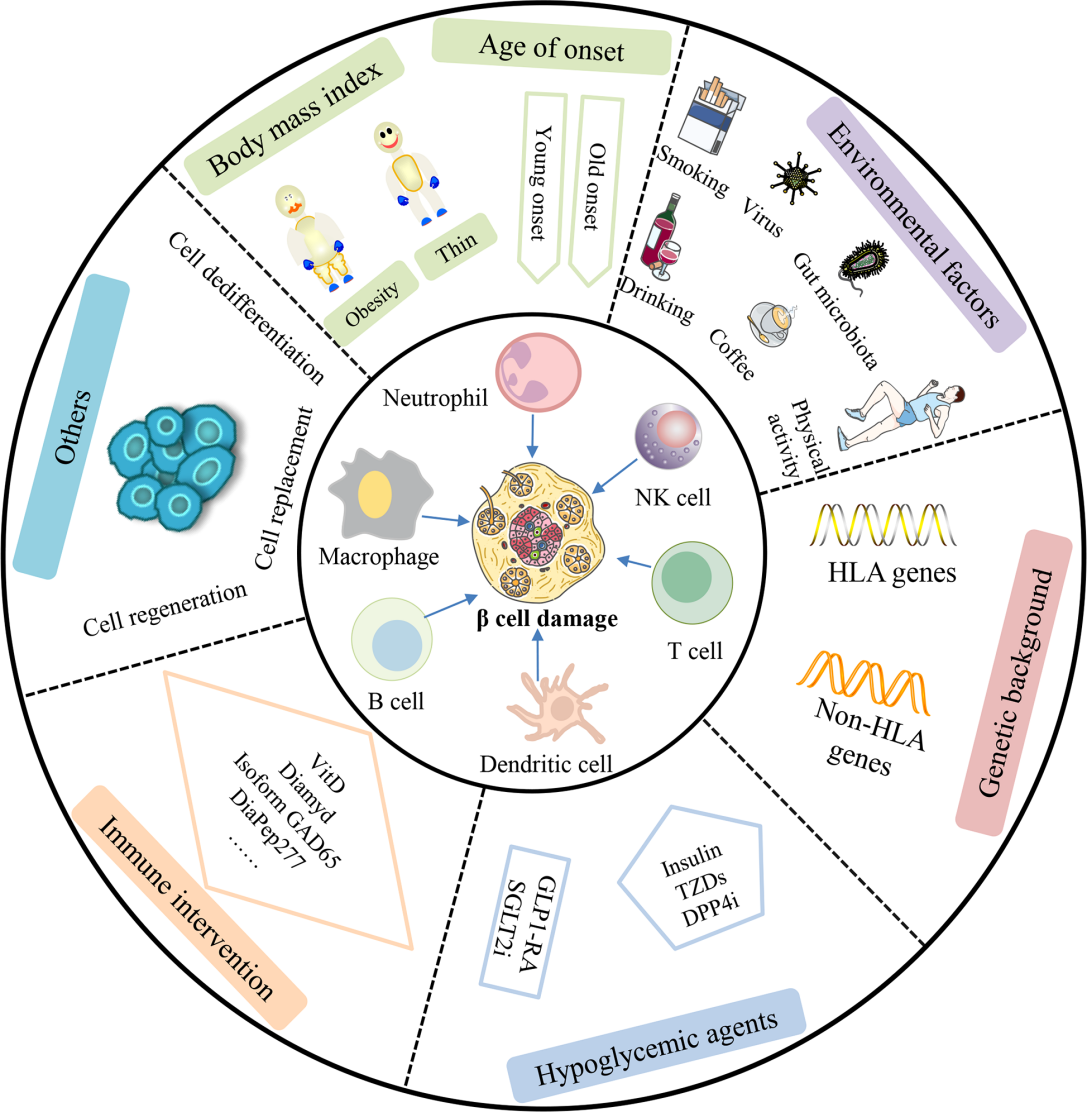
Comprehensive factors affecting β-cell protection and therapy in LADA patients. β-cell function in LADA patients can be affected by cellular, humoral, and innate immunity. Additionally, studies have shown that age of onset, body mass index, environmental factors, genetic background, hypoglycemic agents, and immune intervention can also play an important role in β-cell damage in patients with LADA. In the future, β-cell dedifferentiation, regeneration, and β-cell replacement may be a promising approach to cure LADA patients. NK cell, natural killer cell; TZDs, thiazolidinediones; DPP4i, dipeptidyl-peptidase-4 inhibitor; GLP1-RA, glucagon-like peptide-1 receptor agonist; SGLT2i, sodium-glucose cotransporter 2 inhibitor.

Currently, there is an urgent need for an optimal treatment scheme for LADA patients. This scheme should not only achieve good blood glucose control but also actively protect islet β cell cells and their function and delay and prevent complications as long as possible. The importance of therapeutic methods in protecting islet β-cell function in LADA is attracting significant attention. This article highlighted the risk factors and protection aspects of islet β cells in LADA according to the age of onset, BMI, environmental factors, genetic background, immune mechanisms, hypoglycemic agents, and immune therapeutics (**Figure 2**). To date, delaying islet β-cell exhaustion and precision treatment of LADA still face many challenges. Over the past two decades, research has identified multiple immune cell types and soluble factors that destroy insulin-producing β cells. These insights into disease pathogenesis have enabled the development of therapies to prevent and modify LADA. Furthermore, research concentrating on β-cell protection treatment options can provide potential opportunities for β-cell protection and reversal of diabetes, such as β-cell dedifferentiation, regeneration, and β-cell replacement.

There is a great need for additional studies exploring the protection of islet β cells in LADA prevention and treatment. Several studies have supported the development of β-like cells by transplantation *in vitro* (133). Human pluripotent stem cell-derived pancreatic islets have received widespread attention as a promising cellular resource for diabetes therapy. A recent study has indicated that transoral injection of human chemo-induced pluripotent stem cells into non-human primates with diabetes can significantly recover the secretion of endogenous insulin and improve glycemic management (134). Utilizing current evolving and extensive technologies and platforms to study human β cells will permit a more elaborate investigation of the underlying mechanisms and will also stimulate the further advancement of therapeutic methods dedicated to human β-cell quality and function (135). The progress in stem cell biology and transplant site engineering has provided both innovative sources of cellular material and improved transplantation approaches. Frontier methods to guard against xenograft rejection and relapsing autoimmunity are also included (136). However, there is still a long distance to cross to the application of these to the therapy of β cells in diabetic patients.

## Author contributions

WY performed the paper search and wrote the first draft of the manuscript. SL, ZX, ZZW, and BL critically revised the text and provided substantial scientific contributions. SL and ZGZ proposed the project and revised the manuscript. SL is the guarantor of this work; thus, SL had full access to all study data and took responsibility for the integrity of the data and the accuracy of the data analysis. All authors contributed to the article and approved the submitted version.

## Funding

This study was supported by the Hunan Province Natural Science Funds for Distinguished Young Scholars (Grant No. 2020JJ2053), the key projects supported by the Hunan Health Commission (Grant No. 202103060904), and the independent exploration and innovation projects for postgraduate of Central South University (Grant Nos. 2021zzts1052, 2021zzts0365).

## Conflict of interest

The authors declare that the research was conducted in the absence of any commercial or financial relationships that could be construed as a potential conflict of interest.

## Publisher’s note

All claims expressed in this article are solely those of the authors and do not necessarily represent those of their affiliated organizations, or those of the publisher, the editors and the reviewers. Any product that may be evaluated in this article, or claim that may be made by its manufacturer, is not guaranteed or endorsed by the publisher.
